# Loss of Function of Vasoactive-intestinal Peptide Alters Sex Ratio and Reduces Male Reproductive Fitness in Zebrafish

**DOI:** 10.1210/endocr/bqae082

**Published:** 2024-07-10

**Authors:** Yang Yu, Sakura Tanaka, Ten-Tsao Wong, Yonathan Zohar, Nilli Zmora

**Affiliations:** Department of Marine Biotechnology, Institute of Marine & Environmental Technology, University of Maryland Baltimore County, Baltimore, MD 21202, USA; Department of Marine Biotechnology, Institute of Marine & Environmental Technology, University of Maryland Baltimore County, Baltimore, MD 21202, USA; Department of Marine Biotechnology, Institute of Marine & Environmental Technology, University of Maryland Baltimore County, Baltimore, MD 21202, USA; Department of Marine Biotechnology, Institute of Marine & Environmental Technology, University of Maryland Baltimore County, Baltimore, MD 21202, USA; Department of Marine Biotechnology, Institute of Marine & Environmental Technology, University of Maryland Baltimore County, Baltimore, MD 21202, USA

**Keywords:** Vip, reproduction, testis, steroidogenesis, teleost

## Abstract

Vasoactive-intestinal peptide (Vip) is a pleiotropic peptide with a wide range of distribution and functions. Zebrafish possess 2 isoforms of Vip (a and b), in which Vipa is most homologous to the mammalian form. In female zebrafish, Vipa can stimulate LH secretion from the pituitary but is not essential for female reproduction, as *vipa*^−/−^ females display normal reproduction. In contrast, we have found that *vipa*^−/−^ males are severely subfertile and sex ratio of offspring is female-biased. By analyzing all aspects of male reproduction with wild-type (WT) males, we show that the testes of *vipa*^−/−^ are underdeveloped and contain ∼70% less spermatids compared to WT counterparts. The sperm of *vipa*^−/−^ males displayed reduced potency in terms of fertilization (by ∼80%) and motility span and duration (by ∼50%). In addition, *vipa*^−/−^ male attraction to WT females was largely nonexistent, indicating decreased sexual motivation. We show that *vipa* mRNA and protein is present in Leydig cells and in developing germ cells in the testis of WT, raising the possibility that endogenous Vipa contributes to testicular function. Absence of Vipa in *vipa*^−/−^ males resulted in downregulation of 3 key genes in the androgen synthesis chain in the testis, *3β-hsd, 17β-hsd1*, and *cyp11c1* (*11β-hydrogenase),* associated with a pronounced decrease in 11-ketotestosterone production and, in turn, compromised reproductive fitness. Altogether, this study establishes a crucial role for Vipa in the regulation of male reproduction in zebrafish, like in mammals, with the exception that Vipa is also expressed in zebrafish testis.

Reproduction in vertebrates is regulated by the brain-pituitary-gonadal axis, in which internal and external cues are translated into endocrine signals that control various aspects of reproductive performance. The main axis is governed by the hypothalamic GnRH neurons that induce secretion of LH and FSH from the pituitary. LH and FSH, in turn, reach the gonads and regulate sex steroid synthesis and secretion (1). Because GnRH was found to be dispensable for reproduction in zebrafish (2, 3), and based on transcriptomic analysis of wild-type (WT) and knockout (KO) brains (unpublished), we examined vasoactive intestinal peptide a (Vipa) as a potential factor regulating Lh in female zebrafish (4).

Vip, a 28-amino acid neuropeptide, was initially isolated from the porcine gastrointestinal wall in 1972 (5). Vip is synthesized in various tissues, including the gut, pancreas, cortex, and suprachiasmatic nuclei (SCN) of the hypothalamus in the brain (6-8). This pleiotropic neurohormone is known for its wide-ranging impacts such as stimulating contractility in the heart, inducing vasodilation, triggering glycogenolysis, reducing arterial blood pressure, and relaxing the smooth muscle of the trachea, stomach, and gallbladder (9).

Vip plays a key role in male reproduction. Direct corpus cavernosum injection of Vip, alone or in combination with acetylcholine, has been shown to alleviate functional impotence through the stimulation of nitric oxide in mice (10, 11). In the testis, Vip is released near Leydig cell nests by specific Vip-ergic fibers that are abundant in the inferior spermatic nerve (12). In fact, studies have reported the presence of Vip in axons penetrating the testis in various animals (12-14). In vitro treatment of cultured Leydig cells with Vip resulted in dose-dependent increases in the production of testosterone, progesterone, and pregnenolone (15), suggesting a direct stimulatory effect of Vip on Leydig cells. Vip-deficient males displayed lower FSH and testosterone levels in the serum, which is associated with early signs of testicular degeneration in mice (16).

In mice, Vip neurons in the SCN innervate GnRH neurons in the median preoptic area and convey circadian rhythm to the reproductive axis (17). Although the mechanism underlying Vip action on the testis in mammals is not fully clear, it was suggested that Vip originating from the SCN plays a role because circadian rhythm is perturbed in Vip null mice (18). As a result, the rhythmic secretion of gonadotropins is disrupted in Vip KO mice and, in turn, so are testicular functions (18).

Zebrafish possess 2 isoforms of Vip, Vipa and Vipb, in which Vipa peptide is most homologous to the mammalian Vip (eg, mouse Vip shares 92% homology with zebrafish Vipa). *vipa* gene was shown to be expressed in several brain regions of zebrafish, such as the anterior parvocellular preoptic nucleus, the postcommissural nucleus of the ventral telencephalic area, and the ventromedial thalamic nucleus of the hypothalamus (4). Vipa neurons directly innervate Lh and Fsh gonadotropes in the pituitary of mature females and Vipa peptide intracerebroventricular injection stimulated Lh secretion independent of Gnrh3 (4). Surprisingly however, this study also found that *vipa*^−/−^ and *vipa*^−/−^*/gnrh3*^−/−^ mature ZF females displayed normal reproductive performance and were fertile.

In contrast, and for the first time in a teleost, we have found that reproductive fitness was profoundly and detrimentally affected by the lack of Vipa in *vipa*^−/−^ males. In addition, sex ratio in *vipa*^−/−^ offspring was biased toward females. Consequently, we set out to determine how the lack of Vipa transpired into subfertility and feminization at the level of the gonads.

Zebrafish male reproduction includes a typical mating behavior that precedes the sperm release. During courtship, three initiatory mating activities (chase, tail-nose, and approach) are displayed by both males and females (19, 20). Both courtship and gamete quality are fundamental for successful spawning. Spermatogenesis begins with differentiating germ cells that undergo transcriptional reprogramming and maturation through a diversity of cell types, with support of somatic cells (21, 22). Mammals and zebrafish share an overall testis architecture featuring several tightly coiled seminiferous tubules (23). In the zebrafish testis, Sertoli cells surround individual undifferentiated spermatogonia, where germ cells first develop in a clonal syncytium of type A spermatogonia before undergoing mitotic divisions as type B spermatogonia and then subsequently entering meiosis (24). This developmental sequence is followed by a spermiogenic phase, where spermatids develop into spermatozoa by nuclear condensation, organelle elimination, and formation of the flagellum. The cyst then opens to release mature spermatozoa in the lumen of seminiferous tubules (23). Sertoli cells and Leydig cells produce testosterone and Igf3, which promote spermatogenesis (25, 26).

To understand what causes subfertility in the absence of Vipa in male zebrafish, we examined local expression and presence of Vipa peptide in the testes of WT males and determined the effect of lack of Vipa on mating behavior, sperm potency, sperm quality, and the androgen synthesis pathway. Our results suggest that endogenous testicular Vipa, which is expressed in developing spermatogonia and in Leydig cells, may also contribute to sperm quality, probably by regulating testosterone/11-ketotestosterone (11-KT)-producing enzymes.

## Materials and Methods

### Animal Husbandry

All zebrafish (Tuebingen) were maintained in a 28 °C recirculating aquaculture system with a 14-hour light and 10-hour dark photoperiod. The *Vipa^−/−^* zebrafish line was previously established in our laboratory (4). Before tissue sampling, fish were anesthetized to full sedation using tricaine methanesulfonate (MS-222; Sigma-Aldrich) and rapidly decapitated. The Institutional Animal Care and Use Committee at the University of Maryland School of Medicine approved all experimental protocols (institutional approval #0519010 and #0522013).

### Sex Ratio

WT and *vipa*^−/−^ zebrafish were in-crossed and offspring were raised to adulthood. After genotyping using PCR (4), sex ratios from each spawn were determined for each genotype at age 3 months based on external morphology. The sex of representative females and males was further verified through gonadal inspection and histology.

### Masculinization of Vipa^−/−^ Offspring

To test whether the female-biased sex ratio of the *vipa*^−/−^ offspring can be rescued by testosterone, *vipa*^−/−^ zebrafish larvae from 4 pairs were treated with 0.17 µM methyl-testosterone or ethanol vehicle in fish water for 15 days from 5 to 20 dpf, following a previously published protocol (27). The fish were reared in a spawning tank at 28 °C, with half of the incubation water volume replenished every other day. At 3 months of age, the sex of each individual was determined based on external morphologies and through spawning trials. The sex of representative females and males was further verified through gonadal inspection and histology.

### In Vitro Fertilization

WT female and WT or *vipa*^−/−^ males were paired for spawning the night before, following routine procedure. Sperm (1-2 µL) was collected in the morning by gently stroking the sides of the fish around the urogenital region between the thumb and forefinger, followed by gentle suction with a 2-µL pipette at the urogenital opening, and stored in 50 µL pf Hank's Balanced Salt Solution on ice. Subsequently, eggs were collected by gentle stripping from WT females. The sperm from each *vipa*^−/−^ and WT males were added to the same number of oocytes from eight different WT females in separate 60-mm culture plates. Five milliliters of fish water were added, and the fertilization process was initiated at 28 °C with gentle agitation at 30 rpm. Fertilization rates were quantified after 24 hours by counting fertilized and unfertilized eggs.

### Fertilization Potency

Five-month-old *vipa*^−/−^ and WT siblings were mated with the following combinations: 14 *vipa*^−/−^ males and 10 *vipa*^−/−^ females with WT counterpart and WT males with WT females. In each of the mating combinations, 1 female and 1 male were set for spawning in standard zebrafish spawning tanks with the sexes separated by a divider, which was removed immediately after lights-on at 09:00 Am for a 2-hour period to incite mating. Eggs were collected from each breeding chamber and incubated in fish water at 28 °C. Fecundity (spawned eggs/body weight) and fertilization rate (number of fertilized eggs/total eggs oviposited) were quantified by counting fertilized and unfertilized eggs at 24 hpf, survival rate was determined by the number of hatched embryos at 48 hpf.

### Male to Female Attraction

Male *vipa^−/−^* and WT approaches toward a female were tracked. Six males of each genotype group were used for the assay. To minimize variations, all animals are age- and size-matched at 4 months. A male from either genotype, along with a WT female, were placed in the same chamber, separated by a plastic center divider 20 hours before the actual assay. The next morning, the pairs were transferred to a modified container with a narrow compartment that limited fish movement (3 cm) holding a WT female or male (as a control for sexual attraction). The tested male was placed in the larger 20-cm compartment, which allows free swimming. Immediately after transferring the animals to the assay chamber, the tested male behavior was recorded for 10 minutes by a GoPro Hero 8 camera. The number of male approaches toward the divider was counted, and all movements were tracked using idTracker software (28).

### Sperm Quality

WT or *vipa*^−/−^ males were anaesthetized with MS-222. The genital area of the male was gently pat-dried before 1 to 2 µL of sperm was collected directly from the genital opening using a 2 µL pipette tip and transferred into 50 μL Hank's solution and stored on ice. All sperm assays were performed within 15 minutes after the collection. The diluted sperm was activated by adding 50 μL of fish water at room temperature. Then 5-μL activated sperm solution was then loaded on a semen analysis slide with a sperm counting grid (10 µL w/Grid-Disposable Makler, CellVision) and immediately filmed for 10 minutes using the Olympus BX60 Fluorescence Microscope and Volocity 6.2.1 system, followed by analysis with Volocity 6.2.1 software (https://www.volocity4d.com) and CASA software (https://github.com/calquezar/OpenCASA) (29).

### Gonadal Histology via Hematoxylin and Eosin Staining

Testes were sampled by dissection from sexually mature WT and *vipa*^−/−^ males at 5 months of age and fixed in 4% paraformaldehyde (PFA) in PBS at 4 °C overnight. The testes were transferred into glass vials containing 30% sucrose in 0.1 M phosphate buffer at 4 °C and allowed to completely submerge. After mounting with optimum cutting temperature resin (Sakura Fineteck, Inc.), the testes were sectioned sagittally at a 5-µm thickness using a cryostat (Sakura Fineteck, Inc.), and sections were transferred to positively charged slides (Denville UltraClear; Denville Scientific). The sections were postfixed with 4% PFA and stained with hematoxylin and eosin (H&E) following a standard protocol, dehydrated, mounted, and viewed under Zeiss Axioplan microscope (Carl Zeiss Microscopy).

### In Situ Hybridization

Slides for in situ hybridization (ISH) were prepared similar to H&E staining except for preparing 10-µm-thick sections. The slides were postfixed with 4% PFA, incubated with 1 µg/mL proteinase K (New England Biolabs) in PBS at 37 °C for 10 minutes, and postfixed with 4% PFA in PBS at room temperature for 20 minutes. *Vipa* DIG labeled anti-sense and sense riboprobes were prepared using *vipa* cDNA as a template (GenBank accession #NM_001114553.3) and T7 or Sp6 RNA polymerase. The sections were prehybridized at 62 °C for 2 hours followed by a hybridization with 1000 ng/mL of a generated *vipa* antisense or sense DIG-labeled riboprobe overnight at 62 °C. After washing, sections were blocked with TNB blocking buffer (100 mM Tris-HCl, pH 7.5; 0.15 M NaCl; 0.5% NBT blocking reagent, from Tyramide Signal Amplification kit, TSA; Perkin Elmer). Slides were incubated with anti-DIG Fab fragment conjugated to alkaline phosphatase (Roche), and the hybridization signals were then developed with 4-nitroblue tetrazolium chloride/5-bromo-4-chloro-3-indoyl-phosphate (NBT/BCIP stock solution; Roche). After mounting and counterstaining with nuclear red solution (0.1% nuclear fast red, 5% aluminum sulfate), the slides were imaged using a Zeiss Axioplan microscope (Carl Zeiss Microscopy).

### Immunohistochemistry

Immunohistochemistry was performed on sagittal testes sections (5-micron paraffin sections) from 5-month-old sexually mature WT and *vipa*^−/−^ males. After deparaffinization and rehydration, slides were immersed 15 minutes in 0.1 M citrate buffer, pH 6.0, in a 95 °C water bath for antigen retrieval. Endogenous peroxidases were inactivated with 0.5% H_2_O_2_ in 1 × PBS for 30 minutes at room temperature, and nonspecific background was reduced by incubation in normal goat serum with BSA for 1 hour at room temperature. Sections were then incubated overnight at 4 °C with anti-zfVIPa primary antibodies (raised against the recombinant Vipa precursor, Zohar Yonathan; UMBC Cat# Zohar Lab, RRID:AB_3101965) diluted 1:1000 in PBS with Tween containing 2% normal goat serum as previously described (4), washed, and incubated with goat anti-rabbit antibody conjugated to peroxidase (Perkin Elmer). The signal was revealed using 3,3-diaminobenzidine as the chromogen. Sections were counterstained with hematoxylin solution (Sigma Aldrich). Negative controls were carried out using the preimmune serum from the same immunized rabbits.

### Real-time Quantitative RT-PCR and 11-Ketotestosterone ELISA on Testes

#### Quantitative RT-PCR

Total RNA was extracted from testes of 5-month-old *vipa*^−/−^ and *vipa^+/−^* male siblings using RNAzol reagent, according to the manufacturer's protocol, and total RNA was quantified with a Nanodrop unit (Thermo Scientific). One microgram of total RNA was treated with RQ DNAse 1 at 37 °C and synthesized into first-strand cDNA using a High-Capacity cDNA Reverse Transcription Kit (Invitrogen) in a 20-μL reaction. Quantitative PCR was carried out in duplicate with a final volume of 10 μL: 2 × GoTaq SYBR Green QPCR mix (Promega), 200 nM primer mix, 0.1 × ROX, 40 ng cDNA, and sterile MilliQ water, in a CC7500 Fast Real-Time PCR System (Applied Biosystems, Inc.). Cycling consisted of 40 cycles of 95 °C for 5 and 60 °C for 30 seconds. Levels of *steroidogenic acute regulatory protein* (*StAR)*, *3β-hydroxysteroid dehydrogenase (3β-hsd)*, *17β-hydroxysteroid dehydrogenase (17β-hsd1* and*17β-hsd3),* and *11β-hydroxylase (cyp11b1)* in the testis were determined by amplification using primer sets described elsewhere (30, 31), with *eef1α* as an internal control (Table 1). mRNA levels were normalized using a data-driven normalization algorithm (NORMA-Gene) method (32). The normalization was performed using 7 genes from the same tissue. The algorithm estimates a normalization factor by calculating mean expression values for each replicate for all genes. For each sample, duplicate data are obtained and averaged before normalization, then the fold-change in normalized expression relative to the control was calculated. Biological replicates were averaged to obtain mean fold-change in gene expression ± standard error of mean, as previously described (33).

**Table 1.** Primers used for qPCR assays

Gene	Type	Sequence (5′-3′)	Tm (°C)	GC%
*StAR*	For	ACCTGTTTTCTGGCTGGGATG	65.8 °C	52.00
*StAR*	Rev	GGGTCCATTCTCAGCCCTTAC	65.3 °C	57.10
*3β-HSD*	For	GCAACTCTGGTTTTCCACACTG	62.9 °C	50.00
*3β-HSD*	Rev	CAGCAGGAGCCGTGTAGCTT	65.2 °C	60.00
*17β-HSD1*	For	GGCACCATCCGCACCA	65.3 °C	68.75
*17β-HSD1*	Rev	CTCGTTGAATGGCAAACCCT	63.9 °C	50.00
*17β-HSD3*	For	ATGGTCACATTCACGGCTGA	65 °C	50.00
*17β-HSD3*	Rev	TGCACGATCCTGCCCAG	64.9 °C	64.71
*cyp11c(11β-hydroxylase)*	For	AAGACGCTCCAGTGCTGTG	65.4 °C	57.89
*cyp11c(11β-hydroxylase)*	Rev	CCTCTGACCCTGTGATCTGC	65 °C	60.00
*ef1aα*	For	AAGACAACCCCAAGGCTCTCA	66.7 °C	52.38
*ef1aα*	Rev	CTTTGGAACGGTGTGATTGA	61.5 °C	45.00

Abbreviations: 3β-HSD, 3β-hydroxysteroid dehydrogenase; 17β-HSD1 and 17β-HSD3, 17β-hydroxysteroid dehydrogenase 1 and 3; cyp11c, cytochrome P450, family 11, subfamily c, polypeptide 1 (aka 11β-hydroxylase); ef1aα, elongation factor 1A alpha; GC%, guanine-cytosine percentage; StAR, steroidogenic acute regulatory protein; Tm, melting temperature.

#### 11-KT ELISA

Seven testes were dissected from 3-month-old *vipa*^−/−^ and WT males, respectively, and gonad weight was recorded. Steroid extraction followed the protocol described elsewhere (34). Briefly, the testes were incubated separately in 5 mL 80% methanol at 4 °C for at least 48 hours in low-protein binding tubes (Eppendorf). Testes were then sonicated using a “Branson Sonifier 250’ sonicator equipped with wide probe for 30 seconds at output level 5, centrifuged at 12 000 rpm for 15 minutes at 4 °C, and the supernatant was transferred to a new low protein-binding tube. One milliliter of aliquot of the resulting supernatant was dried in a Speedvac for 2 hours, and then reconstituted with1 mL of ELISA steroid assay buffer.

11-KT levels were determined using 11-KT steroid ELISA reagents kindly provided by Berta Levavi-Sivan following her laboratory's standard protocol (35). A 96-well plate was coated overnight at 4 °C with 150 µL goat anti-rabbit (Sigma R1131) (10 µg/mL in potassium phosphate buffer), then blocked by the addition of 100 µL 1% BSA in potassium phosphate buffer. A total of 50 µL 11-KT standard curve (0-1 ng/mL) and samples were added to each well with 25 µL 11-KT antibody (David E. Kime; The University of Sheffield Cat# Kime DA, RRID:AB_3101966) (36, 37), diluted 1:50 000, and 50 µL 11-KT:AChE Tracer (Cayman 482750), then incubated overnight at room temperature. Plates were subsequently washed 3 times followed by the addition of 200 µL Ellman's reagent and incubated at room temperature for ∼5 hours. Assay results were quantified and analyzed using SpectraMax M5 and SoftMax Pro 5.4 at 405 nm wavelength.

## Results

### Vipa Knockout Males Display Reduced Fecundity and Fertilization Capacity

**Figure 1.**
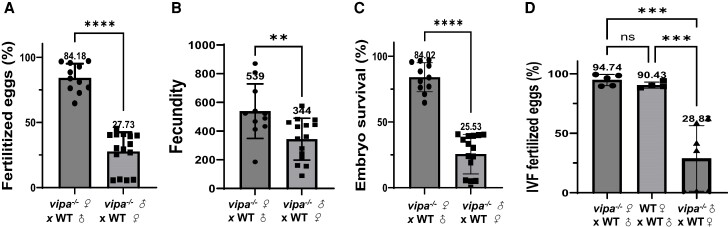
*vipa* ^−/−^ zebrafish males are subfertile. Fertilization, fecundity, and survival rates of embryos produced by crossing *vipa*^−/−^ males with WT females (n = 12) and *vipa*^−/−^ females with WT males (n = 15). (A) Fertilization, (B) fecundity, and (C) survival rates at 48 dpf. Statistical analysis performed using Welch 2-ample *t*-test. (D) Fertilization rates of embryos from in vitro fertilization of sperm from *vipa*^−/−^ males and oocytes from WT females (n = 8), or oocytes from WT females and WT sperm (n = 8). Statistical analysis performed using 1-way ANOVA, followed by Tukey's multiple comparisons test. ****P < .0001, ***P < .001, **P < .01, ns = *P* ≥ .05.

To determine the reproductive fitness of *vipa*^−/−^ zebrafish male, *vipa*^−/−^ males or female zebrafish were crossed with WT counterparts followed by fertility and fecundity tests. The total eggs obtained from 11 pairs of *vipa*^−/−^ females crossed with WT males was 5925, and 15 pairs of *vipa*^−/−^ males crossed with WT females was 5159. The mean fertilization rate for each group: *vipa*^−/−^ female with WT male was 84.18 ± 3.286% and *vipa*^−/−^ male with WT female was 27.73 ± 3.811% (*P* < .0001), fecundities of 538 ± 57 and 343 ± 37 on average per female, respectively (*P* = .0069) (Fig. 1A-C).

To determine whether the low fecundity and fertilization rate were caused by compromised mating behavior, we also performed in vitro fertilization trials, where sperm of *vipa*^−/−^ and WT males was used to fertilize eggs obtained from the same WT female and divided equally. Significantly lower fertilization rates (28.83 ± 10.41%) were recorded for *vipa*^−/−^ sperm compared to WT sperm (90.43 ± 1.28%) (*P* = .0001) or compared to *vipa*^−/−^ eggs fertilized with WT sperm (94.74 ± 2.09%) (*P* = .001383) (Fig. 1D).

### Sex Ratio is Female-biased in Vipa^−/−^ Offspring and is Reversed With Methyl-testosterone

The sex ratio of female to male of *vipa*^−/−^ was consistently biased toward females with an average 81.5 ± 1.06% to 18.5 ± 1.09% female-to-male ratio, compared to a 57 ± 3.39% and 43 ± 3.3% ratio in WT. All females with female external morphologies had ovaries. To understand whether female-biased sex ratio in *vipa*^−/−^ could be reversed by exposure to testosterone, the offspring were subjected to a masculinization procedure using methyl-testosterone. The treatment increased the male prevalence to 74.16 ± 3.27% and 90.16 ± 1.61% and reduced that of females to 25.84 ± 3.27% and 9.84 ± 3.27% for *vipa*^−/−^ and WT, respectively (Table 2).

**Table 2.** Sex is biased toward females in *vipa*^−/−^ offspring and is reversed with androgen treatment

Sex (%)	Treatment			
	Female	*P* value	Male	*P* value
WT (vehicle)	57.5	<.001	42.5	<.001
WT (MT)	9.84	<.001	90.16	<.001
*vipa* ^−/−^ (vehicle)	81.5	<.001	18.5	<.001
*vipa* ^−/−^ (MT)	25.84	<.005	74.16	<.005

Sex ratio of homozygous *vipa*^−/−^ and WT offspring treated with methyl-testosterone or only vehicle. Sex was determined in 3 month-old adults. Statistical analysis performed using 1-way ANOVA, followed by Tukey's multiple comparisons test.

### Vipa^−/−^ Males Exhibit Reduced Attraction to Females

**Figure 2.**
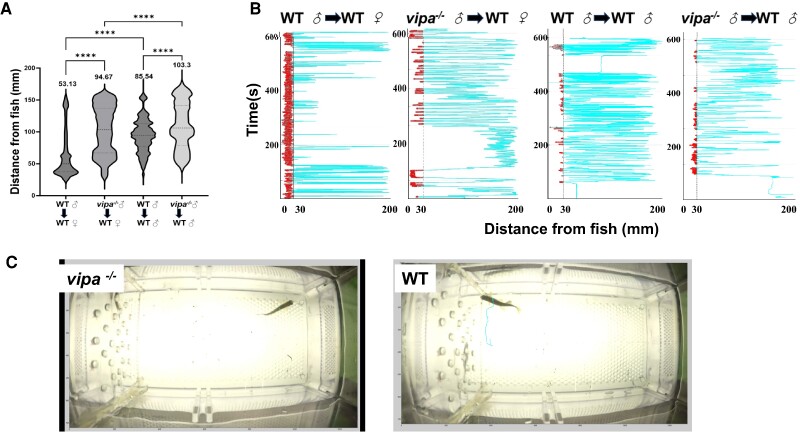
*vipa* ^−/−^ males display reduced attraction to female. *vipa*^−/−^ and WT males’ distance from WT female and male zebrafish and the time spent near confined females or males. (A) Time spent within 30 mm of female/male chambers by *vipa*^−/−^ and WT males (n = 5 males of each type, respectively). (B) Tracking distribution map of a representative male of each type (WT and *vipa*^−/−^) using idTracker software to assess images taken at 30 frames/second intervals. (C) Thirty-second representative tracking videos of a *vipa*^−/−^ (left) and WT male (right) with the same confined WT female (circled). Statistical analysis for (A) was performed on mean values using 1-way ANOVA, followed by Tukey's multiple comparisons test, ****P < .0001.

To investigate the effect of Vipa on male zebrafish sexual and motivation, we tracked the swimming pattern of WT and *vipa*^−/−^ male zebrafish with similarly sized WT female zebrafish or WT male. In general, *vipa*^−/−^ males exhibited less approaches toward the confined fish (described in Methods), regardless of the sex, than WT males. *Vipa*^−/−^ males spent an average of 1.26 ± 0.35 minutes, whereas WT males spent an average of 4.03 ± 1.02 minutes of the recorded 10 minutes within 3 cm distance of the WT female chamber (Fig. 2A) (*P* < .0001). On average, the mean distance from the confined female exhibited by WT and *vipa*^−/−^ was 53 and 94 mm, respectively. This distance grew to 85 and 103 mm, respectively, when a male was placed in the confinement chamber. During the 10-minute recording, *vipa*^−/−^ males wandered away from the divider, whereas WT males often swam into a 30-mm range of the divider (Fig. 2A and 2B).

### Vipa^−/−^ Males Exhibit Smaller and Disorganized Testis and Reduced Sperm Quality

**Figure 3.**
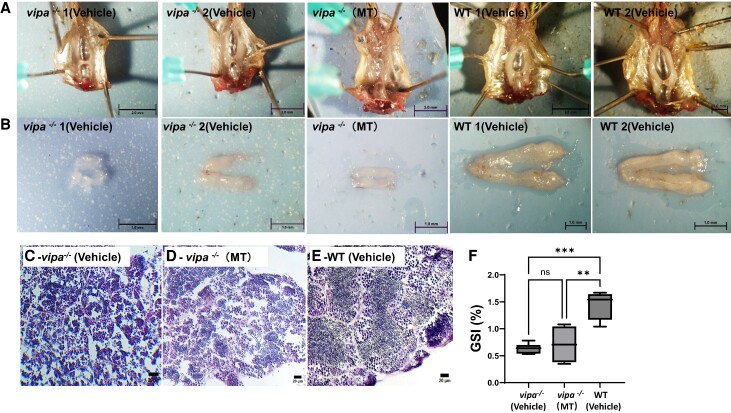
Gonadal morphology and gametogenesis of *vipa*^−/−^ and WT testes. Gross testes morphology (in situ) of 2 representative *vipa*^−/−^ and WT adult males (A; top row), and after excision (B; bottom row). (C) Testis histology with hematoxylin and eosin staining of vehicle-treated *vipa*^−/−^ male. (D) Testis histology with hematoxylin and eosin staining of methyl-testosterone treated *vipa*^−/−^ male. (E) Testis histology with hematoxylin and eosin staining of WT male. (F) Average GSI of WT and *vipa*^−/−^ males (n = 6). Statistical analysis performed using the Welch 2-sample *t*-test. *P* (GSI) = .0092 and .0002, **P < .01, ***P < .001.

**Figure 4.**
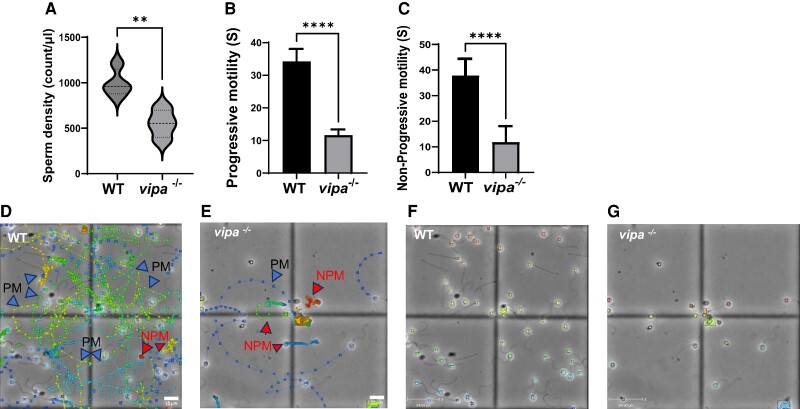
*Vipa* ^−/−^ males display reduced sperm quality (A) average sperm density of WT and *vipa*^−/−^ (*n* = 4). (B) Average sperm motility (n = 8), and (C) average progressive motility (n = 8). (D, E) sperm motility tracking of WT and *vipa*^−/−^ males. Arrows point to either progressive (PM) or nonprogressive (NPM) motility paths. (F, G) Tracking video for *vipa*^−/−^ and WT male sperm. Statistical analysis performed Welch 2-sample *t*-test. *P* (sperm density) = .0062, *P* (PM) < .0001, and *P* (NPM) < .0001. ****P < .0001, **P < .01.

To determine the effect of lack of Vipa on the testis, we examined testis morphology and sperm quality. Gross morphology (Fig. 3A and 3B) and gonado-somatic index (GSI) (Fig. 3F) analysis of testes from WT and *vipa*^−/−^ males revealed significantly (2 times) smaller testes in *vipa*^−/−^ compared to WT. Histological inspection of testicular sections with H&E staining revealed a striking difference between the 2 genotypes: whereas WT testis contained a large number of mature spermatozoa (Fig. 3E), that of *vipa*^−/−^ contained significantly less spermatids with empty spaces throughout the tissue (Fig. 3C and 3D). Sperm quality of WT and *vipa*^−/−^ males was determined by both sperm count and motility using the CASA algorithm (29). Sperm concentration of WT averaged ∼1000/µL, whereas that of *vipa*^−/−^ males was significantly (50%) lower, averaging ∼500/µL (Fig. 4A). Progressive motility (rapid straight or large circles) (Fig. 4B) and nonprogressive motility (small tight circles) (Fig. 4C) of *vipa*^−/−^ males lasted about half and one-third the time of WT males, respectively.

### Vipa mRNA and Peptide are Detected in Testis

**Figure 5.**
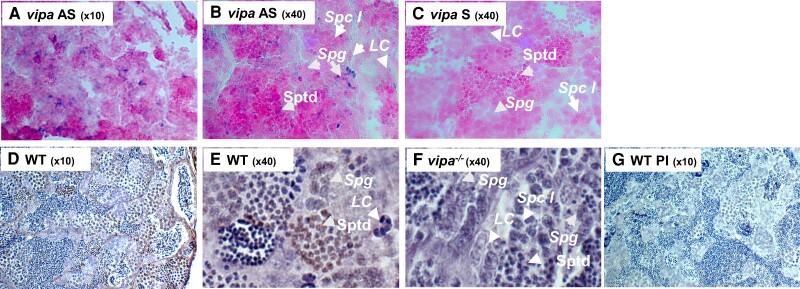
Vipa mRNA and peptide are detected in zebrafish testis. In situ hybridization using vipa antisense riboprobe (A, B) and sense riboprobe (C) on sections from WT testis. Arrows point to cells where the positive signal was observed with the antisense riboprobe. Immunostaining of WT (D, E) and *vipa*^−/−^ (F) testis with anti-Vipa precursor, and WT with preimmune serum (G). LC, Leydig cells; Ps, prespermatogonia; Spc I, primary spermatocytes; Spg, spermatogonia; Sptd, spermatids.

In light of the profound effect on the testis, we sought to test whether *vipa* is endogenously expressed in the zebrafish testis. Therefore, we examined the presence of *vipa* mRNA using ISH and Vipa peptide via immunohistochemistry in the testes of WT and *vipa*^−/−^ zebrafish. *vipa* mRNA was detected in Leydig cells, spermatocytes A and B, spermatogonia and spermatids (Fig. 5B). Vipa peptide was detected in prespermatogonia, primary spermatocytes, second spermatocytes, Leydig cells, and Sertoli cells in the WT zebrafish testis (Fig. 5D and 5E). Immunostaining of *vipa*^−/−^ testis with the same antibodies did not result in any signal (Fig. 5F). Immunostaining using the preimmune serum did not result in signal on consecutive sections (Fig. 5G).

### Androgenic-related Genes are Downregulated in Vipa^−/−^ Males

**Figure 6.**
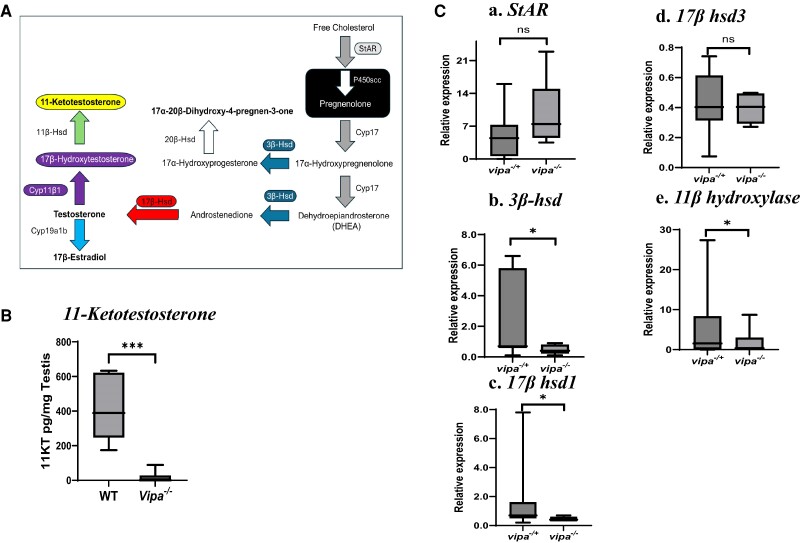
The effect of loss of *vipa* on androgen synthesis and sex steroid hormone levels. (A) Schematic diagram of androgen synthesis pathway in Leydig cells in teleost (Adopted from (63). Each enzyme is signified by a color-coded arrow. (B) 11-Ketotestosterone levels in WT and *vipa*^−/−^ male testes. (C) Comparison of the expression of genes encode for steroid synthesis enzymes in *vipa^−/+^* and *vipa*^−/−^ male siblings. (a) *StAR*, (b) *3β-hsd*, (c) *17β-hsd1*, (d) *17β-hsd3*, (e) 11β-hydroxylase (*cyp11β1* or *cypc1*). Results are presented as mean relative expression ± standard error of mean normalized using NORMA-Gene platform with 7 different genes for the gene expression study. 11-KT levels are presented as pg/mg testis using pairwise analysis with nonparametric statistical analysis. Statistical significance was accepted when *P* ≤ .05.

To determine the effect of lack of Vipa on testicular steroidogenesis/testosterone synthesis, we measured expression of genes encoding enzymes involved in androgenic steroid synthesis (Fig 6A): *Steroidogenic Acute Regulatory Protein* (*StAR)*, *3β-hydroxysteroid dehydrogenase (3β-hsd)*, *17β-hydroxysteroid dehydrogenases (17β-hsd1* and*17β-hsd3),* and *11β-hydroxylase (cyp11b1)* in the testes of *vipa^−/+^* and *vipa*^−/−^ siblings. Although no difference was observed with *StAR* and *17β-hsd3* transcript levels, *3β-hsd*, *17β-hsd1,* and *cyp11c1* were significantly downregulated by 71%, 40%, and 60% in *vipa*^−/−^ compared to *vip^+/−^* testis (*P* ≥ .05), respectively (Fig. 6C).

To determine the effect of lack of Vipa on 11-Ketotestosterone levels, we measured the 11-KT in the testes of WT and *vipa*^−/−^. Levels of 11-KT in WT testes varied from ∼250 to 600 pg/mg testis tissue between the 7 individuals averaging ∼400 pg/mg. 11-KT levels in *vipa*^−/−^ testes were undetectable in 4 of the 7 testes and close to 0 in the other 3 (Fig. 6B).

## Discussion

In the framework of this study, we demonstrated that Vipa-deficient mature zebrafish males are subfertile, indicating that Vipa has an important functional role in the testis. Through a series of tests, we show that sperm quality, potency, and attraction to females are severely compromised in the *vipa*^−/−^ males.

We fir**s**t noticed the subfertility condition during efforts to propagate the *vipa*^−/−^ line. Our attempts to in-cross homozygous mutant fish were largely unsuccessful. Further examinations, which include different combinations of crosses of heterozygous and homozygous *vipa*^−/−^ with WT males and females substantiated our initial observations. In addition, we have encountered great difficulty obtaining a sufficient number of males from the few small spawns obtained from *vipa*^−/−^ parents. Although Dmrt1 (double-sex- and mab-3-related transcription factor 1) and anti-müllerian hormone that promotes male sexual differentiation (38, 39) are established factors, information about the role of Vip in sexual differentiation is scarce. However, 1 study implicated Vip in sexual differentiation of male mice neonates through the mediation of prolactin response to androgens (40). In contrast, to date, there is no strong evidence for the involvement of androgens in sex differentiation in fish (41), including in zebrafish (42). In fact, blocking of endogenous estrogen synthesis in the ovaries induces complete sex reversal to fertile males (42-44). In that regard, it has been shown that administration of methyl testosterone suppresses the expression of all steroidogenic enzyme genes in vitro, including *cyp19a1a* aromatase, which converts testosterone to estradiol (45). Hence, it is possible that the downregulation of *cyp19a1a* is the reason for the masculinization of both WT and *vipa*^−/−^ fingerling observed in our study. Nevertheless, despite our finding that the expression of critical androgenic enzymes and 11-KT levels are reduced in mature *vipa*^−/−^ males, the mechanism underlying Vipa involvement in sex differentiation has yet to be determined.

Vip mRNA levels and distribution in the brain and pituitary display sexual dimorphism. In mammals, including in humans, Vip levels in certain brain regions are higher in males (46, 47), and in the pituitary this dimorphism is associated with the prolactin secretory effect of Vip (48). Interestingly, in the medaka, Vip expression in neurons populating a defined nucleus in the preoptic area also innervate the pituitary in females, whereas pituitary adenylate cyclase-activating polypeptide observed in the same nucleus is found in males (49). Because *fshβ* and *lhβ* double-mutant zebrafish are all males, with delayed testicular development (50), the possibility that the lack of Vipa in the *vipa*^−/−^ male hinders testicular development through its effect on gonadotropins cannot be ruled out. Because our study determined that Vipa is expressed in the testis, Vipa may endogenously regulate testicular function, suggesting a dual regulatory pathway by VIPa. The possibility of a dual brain-pituitary and gonadal regulation by Vip is supported by the fact that VIP is found and exerts its control both at the hypothalamic-hypophyseal level, as well as in the genital organs in mammals (51). Nevertheless, the observation that female zebrafish reproduction is not affected by the lack of Vipa is surprising because the importance of VIP to the function of the ovary is widely documented (52). This may infer that brain-pituitary Vip plays a minor role in the regulation of the gonads in zebrafish.

SCN Vip is 1 of the 2 major neuropeptides mediating circadian rhythms in mammalian brain. SCN Vip neurons directly innervate GnRH neurons and mediate their activity (53). Unlike vertebrates, teleost brain lacks SCN and circadian rhythm is controlled in a decentralized fashion, with all tissues and the majority of cells possessing a direct-light entrainable circadian pacemaker (54-56). Hence, Vipa may function as a circadian clock regulator in the many tissues that express it. Further studies are required to test this possibility.

*Vipa* mRNA and protein are found in the testicular tissue of mature zebrafish males, including in Leydig cells, where steroidogenesis takes place, but also in developing and mature spermatids. Examination of the published transcriptome data from bulk WT zebrafish testis detected transcripts of *vipa* and genes encoding its cognate receptors, ie, *adcyap1b*, *vipr1b*, *vipr2, vpac2r,* and *adcyap1r1*a (https://www.ncbi.nlm.nih.gov/geo/query/acc.cgi?acc=GSM5820552) (57). In mammalian vertebrates, Vip is found in nerves surrounding and penetrating the testis of mice (11, 12, 15), where it induces steroidogenesis. Despite efforts, we could not colocalize Vipa peptide in nerve structures in the testis, as described in mammalian species. Congruently, the presence of Vip was previously shown in mitotic and differentiating germ cells as well as in Leydig, Sertoli cells, prespermatogonia, and spermatogonia of 2 nonmammalian vertebrates, the cartilaginous fish *Torpedo marmorata,* and the wall lizard *Podarcis sicula* (58, 59). These differences in the testicular expression pattern of *vipa* between mammalian and nonmammalian vertebrates may represent evolutionary projection of this gene, (ie, from broad expression in the ray [Elasmobranchii], to a limited expression in zebrafish [Cypriniformes] and no endogenous expression in mammalian species).

We have established that *vipa*^−/−^ male subfertility is caused by poor sperm quality characterized by lower sperm count, reduced motility range and span, as well as lack of attraction to females. *vipa*^−/−^ males displayed a pronounced reduction in their attraction to females compared to WT males, but surprisingly also slightly lower attraction when compared to *vipa*^−/−^ male to WT female or WT male. This may suggest that the absence of Vipa affects social behavior to a lesser degree.

Both sexual behavior and sperm quality are highly dependent on testosterone and 11-KT levels (60). Although the expression levels of the steroid synthesis pathway-related genes tested in the heterozygous *vipa^−/+^* males were highly variable between individuals, these variations were significantly reduced in the *vipa*^−/−^ cohort. This pattern of high diversity of gene expression in the testis has been described in other studies (61, 62), and is probably a common phenomenon for some genes. Indeed, we have found that gene expressions of *3β-hsd*, *17β-hsd1,* and *11β hydroxylase* are downregulated in *vipa*^−/−^ testis, which implies that levels of testosterone, dihydroxytestosterone and 11-KT may, in turn, be depleted (63). Congruently, and unlike in WT testes, 11-KT levels in the *vipa*^−/−^ testes were significantly reduced and were often undetectable, thus supporting the idea that androgen synthesis is compromised in *vipa*^−/−^ testis. Of the 3 enzymes, 17β-hsd1, which converts androstenedione to T, is required for male mouse fertility (64), whereas the essentiality of 3β-hsd, which catalyzes the biosynthesis of progesterone from pregnenolone, 17α-hydroxyprogesterone from 17α-hydroxypregnenolone, and androstenedione from dehydroepiandrosterone, in male reproduction is controversial (65). Moreover, *11* β*-hydroxylase* and *ferredoxin 1*β male mutant zebrafish, the latter of which is required for androgen synthesis, exhibited secondary female sex characteristics associated with lower androgen levels (31, 62). Unlike *vipa*^−/−^, these males had viable sperm when tested in vitro. However, in contrast to the findings in (29, 52), we have found that all *vipa*^−/−^ females had only ovaries, implicating Vipa in the development of male secondary sex characteristics. The differences between lower androgen levels resulting from 11 β-hydroxylase and ferredoxin 1b deficiency, which directly impact androgen levels, and the Vipa mutant, which also features Vipa deficiency, suggest that Vipa has an additional non-steroidogenic effect in the testis. As added support, incubation of human sperm with the VIP peptide increased its motility and the concentration of motile sperm beyond the steroid-dependent phase (66). The likelihood that Vipa plays several roles in the testis is supported by the presence of Vipa not only in the steroidogenic Leydig cells, but also developing and mature spermatids (at various stages of development). Interestingly, androgen receptor-deficient zebrafish males are strikingly similar phenotypically to the *vipa*^−/−^ male (eg, smaller testis size, infertility when tested by natural mating, and only a small amount of mature spermatozoa with a lower fertilization capacity by in vitro fertilization) (67). This suggests that loss of function of a single androgen synthesis gene results in a moderate response compared to the more dramatic disruption caused by the inactivation of the androgen receptor. When taken collectively, the information signifies that Vipa has a comparable importance to that of the androgen receptor.

This study demonstrates the importance of Vipa to male reproduction and male sex differentiation in zebrafish. We showed that lack of Vipa results in downregulation of 3 major enzymes in the synthesis chain of androgens in the testis, which may in turn decrease testosterone production. The putative lower testosterone and 11-ketotestosterone levels may explain the biased sex ratio, as well as the underdeveloped testes and lack of sexual motivation in *vipa*^−/−^ males. However, because lower androgen levels do not hamper sperm quality, Vipa may contribute to testicular function via other pathways. Further studies are required to determine whether the source of Vipa is solely the testis or a combination of sources that includes brain/circulation sources and testicular production.

## Data Availability

Original data generated and analyzed during this study are included in this published article.

## Abbreviations

11-KT: 11-ketotestosterone
H&E: hematoxylin and eosin
ISH: in situ hybridization
KO: knockout
PFA: paraformaldehyde
SCN: suprachiasmatic nuclei
Vipa: vasoactive intestinal peptide a
WT: wild-type
